# Study on Closely Related *Citrus* CMMs based on Chemometrics and Prediction of Components-Targets-Diseases Network by Ingenuity Pathway Analysis

**DOI:** 10.1155/2022/1106353

**Published:** 2022-03-31

**Authors:** Qixuan Mu, Yaping Zhang, Ying Cui, Xin Chai, Junlian Liu, Yongzhi Li, Huijuan Yu, Yuefei Wang

**Affiliations:** ^1^State Key Laboratory of Component-based Chinese Medicine, Tianjin Key Laboratory of TCM Chemistry and Analysis, Tianjin University of Traditional Chinese Medicine, Tianjin 301617, China; ^2^China Astronaut Research and Training Center, Beijing 100094, China

## Abstract

As the representatives of closely related Chinese medicinal materials (CMMs) originated from Rutaceae family, *Aurantii fructus immaturus* (AFI), *Aurantii fructus* (AF), *Citri reticulatae pericarpium viride* (CRPV), and *Citri reticulatae pericarpium* (CRP) have better functions in regulating qi and promoting gastrointestinal motility. However, differences in the quality of closely related *Citrus* CMMs have not yet been revealed until now. Herein, this study focused on the systematic differentiation and in-depth understanding of closely related *Citrus* CMMs by a strategy integrating chemometrics and network pharmacology. Determined by ultra performance liquid chromatography, the content of nine flavonoids showed obvious fluctuations in the decoction pieces from different species (*Citrus aurantium Linnaeus* and *Citrus reticulate Blanco*) with decreasing levels in the samples of ripe fruits. Decoction pieces from the different species and ripening stages were well distinguished by orthogonal projection to latent structure-discriminate analysis (OPLS-DA) and cluster analysis. As a result, four active components including narirutin, naringenin, hesperidin, and 3,5,6,7,8,3′,4′-heptemthoxyflavone were filtered out by variable importance for the projection (VIP) value (VIP > 1.0), which were regarded as chemotaxonomic markers. Furthermore, a components-targets-diseases network was constructed via ingenuity pathway analysis (IPA), and the correlations were predicted between four chemotaxonomic markers, 223 targets, and three diseases including colitis, breast cancer, and colorectal cancer. The obtained results will be of great significance for identifying closely related *Citrus* CMMs and conduce to improving the resource utilization of CMMs.

## 1. Introduction

*Citrus* fruits, known as medicinal and edible homologous plants, have played a vital role in traditional Chinese medicine (TCM) practices and have been extensively applied for thousands of years owing to their greater biological activities, abundant resources, and low toxicity [1]. The Chinese Pharmacopeia stipulates that the fruit of *Citrus aurantium Linnaeus* or its cultivars harvested in May and June should be used as *Aurantii fructus immaturus* (AFI), and those harvested in July are employed as *Aurantii fructus* (AF); besides, the pericarp of *Citrus reticulate Blanco* or its cultivars harvested during August to December should be treated as *Citri reticulatae pericarpium* (CRP), and those harvested in July should be used as *Citri reticulatae pericarpium viride* (CRPV) [2]. Due to their specific therapeutic effects, four decoction pieces are employed to perform diverse usages. AFI is often used to eliminate phlegm and dissipate stagnant qi, while AF relieves gastrointestinal indigestion in a gentle yet effective manner [3, 4]. CRPV is mostly used to promote the flow of liver qi, while CRP is commonly utilized to strengthen the spleen and dispel phlegm [5, 6]. However, the different chemical features in the four decoction pieces have not been systematically reported, which is not beneficial for understanding the different clinical applications.

Understanding the correlation between components and their efficacy contributes to the rational clinical application of CMM. Network pharmacology is an ideal method that uses bioinformatics to predict the action mechanism of herbal ingredients through strategies of multicomponents and multitargets [7]. It is usually applied to clarify the pharmacodynamic basis and mechanisms of CMM, such as Ginseng [8], Licorice [9], and Huangqi [10]. Network pharmacology can predict the relationships between components and diseases, which will help to better understand the differences in quality of the four closely related *Citrus* CMMs.

At present, related reports mainly focus on the analysis of chemical composition and the content of closely related *Citrus* CMMs by employing high-performance liquid chromatography (HPLC) [11–13], liquid chromatography-mass spectrometry (LC-MS) [14–17], and gas chromatography-mass spectrometry (GC-MS) [18, 19]. In addition, researchers have established multiple strategies for distinguishing closely related *Citrus* CMMs based on qualitative or quantitative data with chemometric methods. Yi et al. revealed the volatile chemical features of CRP and CRPV by combing GC-MS metabolomics analysis with chemometrics [20]. Li et al. established a comprehensive strategy for the comparison of AFI and AF by integrating HPLC/GC-MS analysis and chemometric methods [21]. According to UPLC-Q-TOF-MS-based metabolomics, Zhao et al. depicted variable chemotaxonomic markers and metabolic mechanisms of four closely related *Citrus* CMMs [22]. These methods can successfully discriminate the closely related *Citrus* CMMs with the merits of high sensitivity and excellent specificity, facilitating a better understanding of their different medicinal values. Nevertheless, the reported methods suffer from some limitations, such as the limited number of focused compounds for quantitative analysis, an imperfection in the integration analysis of quantitative analysis-chemometrics-network pharmacology for discriminating four closely related *Citrus* CMMs.

Herein, a comprehensive strategy integrating the quantification of nine flavonoids and chemometrics analysis was firstly proposed to identify the four closely related *Citrus* CMMs. This study focused on the analysis of major flavonoids in AFI, AF, CRP, and CRPV through UPLC-variable wavelength detection method. Different species (C. *aurantium* L. and C. *reticulate Blanco*) and development stages (AFI and AF, CRPV and CRP) were clearly discriminated via orthogonal signal correction partial least squares-discriminate analysis (OPLS-DA) and cluster analysis. By variable importance for the projection (VIP) value, narirutin (Nar), hesperidin (Hed), naringenin (Nag), and 3,5,6,7,8,3′,4′-heptemthoxyflavone (Hep) were identified as potential chemotaxonomic markers. Furthermore, ingenuity pathway analysis (IPA) was applied to predict the correlation between four chemical markers and three diseases including colitis, breast cancer, and colorectal cancer. In conclusion, this research aims to offer a simple and convenient strategy for better understanding the different characteristics of four closely related *Citrus* CMMs.

## 2. Materials and Methods

### 2.1. Reagents and Materials

Methanol and acetonitrile (HPLC grade) were manufactured by Sigma-Aldrich Corp. (St. Louis, MO, USA). Formic acid was purchased from Shanghai Aladdin Bio-Chem Technology Co. Ltd (Shanghai, China). Water was purified by a Milli-Q water purification system (Millipore, Billerica, MA, USA). Reference standards, including narirutin (Nar), naringin (Nan), hesperidin (Hed), neohesperidin (Ned), naringenin (Nag), nobiletin (Not), 3,5,6,7,8,3′,4′-heptemthoxyflavone (Hep), and tangeretin (Tar), were obtained from Shanghai Yuanye Biotechnology Co. Ltd (Shanghai, China). Poncirin (Por) was obtained from Shanghai Standard Biotech Co. Ltd (Shanghai, China).

Eighty batches of samples including 20 batches of AFI (numbered AFI1–AFI20), 20 batches of AF (numbered AF1–AF20), 20 batches of CRP (numbered CRP1–CRP20), and 20 batches of CRPV (numbered CRPV1–CRPV20) were bought from the medicinal materials markets, whose detailed information is shown in Table S1. All samples were deposited in State Key Laboratory of Component-based Chinese Medicine, Tianjin University of Traditional Chinese Medicine (Tianjin, China).

### 2.2. Preparation of Standard Solution

Nine standard components were accurately weighted and directly dissolved in methanol to obtain nine stock solutions with concentrations at 4.148 mg/mL for Nar, 11.998 mg/mL for Nan, 7.002 mg/mL for Hed, 10.006 mg/mL for Ned, 2.416 mg/mL for Por, 1.505 mg/mL for Nag, 1.511 mg/mL for Not, 0.051 mg/mL for Hep, and 0.559 mg/mL for Tar, respectively. Afterward, a mixed solution was prepared with the final concentrations of 0.581 mg/mL for Nar, 1.320 mg/mL for Nan, 0.572 mg/mL for Hed, 1.301 mg/mL for Ned, 0.338 mg/mL for Por, 0.211 mg/mL for Nag, 0.060 mg/mL for Not, 0.003 mg/mL for Hep, and 0.050 mg/mL for Tar, respectively. Subsequently, the working solution was diluted into a series of standard solutions with different concentrations to build calibration curves.

### 2.3. Preparation of Sample Solution

Accurately weighed sample powder (0.2 g) was transferred into a conical flask and sonicated with 30 mL methanol at 60°C for 30 min. After centrifugation (18,213 g, 10 min), the supernatant was injected into the UPLC system for detecting Nar, Nan, Hed, Ned, Por, Nag, Not, Hep, and Tar. Only for AFI and AF, the supernatant was subjected to diluting five times with methanol for the analysis of Nan and Ned.

### 2.4. UPLC Analysis

Quantification of the focused compounds from samples was executed on ACQUITY UPLC H-class plus system (Waters Corporation, Milford, MA, USA). 0.2% formic acid aqueous solution (A) and acetonitrile (B) were employed to perform excellent separation on ACQUITY UPLC^®^ BEH C_18_ Column (100 mm × 2.1 mm, 1.7 *μ*m) at 35°C. The flow rate was 0.3 mL/min. The gradient elution program was employed as follows: 19–25% B in 0–4 min, 25–60% B in 4–11 min, and 60–90% B in 11–15 min. Different detection wavelengths were performed accordingly for detecting the focused compounds, with 280 nm in 0–9 min for Nar, Nan, Hed, Ned, Por, and Nag, and 330 nm in 10–15 min for Not, Hep, and Tar. The sample injection volume was 2 *μ*L.

### 2.5. Methodological Validation

For testing the feasibility of the analytical method for quantitative analysis of the tested compounds, precision (intra- and interday), stability, linearity, limit of detection (LOD), limit of quantification (LOQ), reproducibility, and recovery were systematically validated. Intraday and interday precision were evaluated by performing six replicate injections on the same day and three consecutive days, respectively. The stability of the sample solutions prepared at room temperature was assessed by repeated injections at 0, 2, 4, 6, 8, 10, and 12 h, respectively. Two replicate calibration curves were established based on the peak area (*y*) and the corresponding concentration (*x*) of the tested compounds. LOD and LOQ were calculated at a signal-to-noise ratio (S/N) of about 3 and 10 using standard solutions, respectively. Six sample solutions extracted from the same batch were analyzed to confirm repeatability. The recovery was investigated by analyzing six sample solutions processed by adding appropriate amounts of standard solution to 0.1 g sample powder.

### 2.6. Ingenuity Pathway Analysis

The structures of the four tested compounds including Hep, Nar, Hed, and Nag were downloaded from the PubChem database (https://pubchem.ncbi.nlm.nih.gov/) [23]. Potential human targets of the four components were obtained from the Swiss Target Prediction database (http://www.swisstargetprediction.ch/) [24]. All targets were imported into Microsoft Excel software to remove duplicate values. IPA was employed to predict canonical pathways and networks of compounds, targets, and diseases based on known interactions between genes and proteins.

### 2.7. Data Analysis

The Originpro 2016 software (Originlab Corp. Northampton, MA, USA) and SIMCA 14.1 software (Umetrics, Umea, Sweden) were used for the statistical analysis of data. Duplicate targets were removed by Microsoft Excel 2016 (Microsoft Corp., Redmond, WA, USA).

## 3. Results and Discussion

### 3.1. Optimization of Extraction Conditions and Methodology Validation

**Figure 1:**
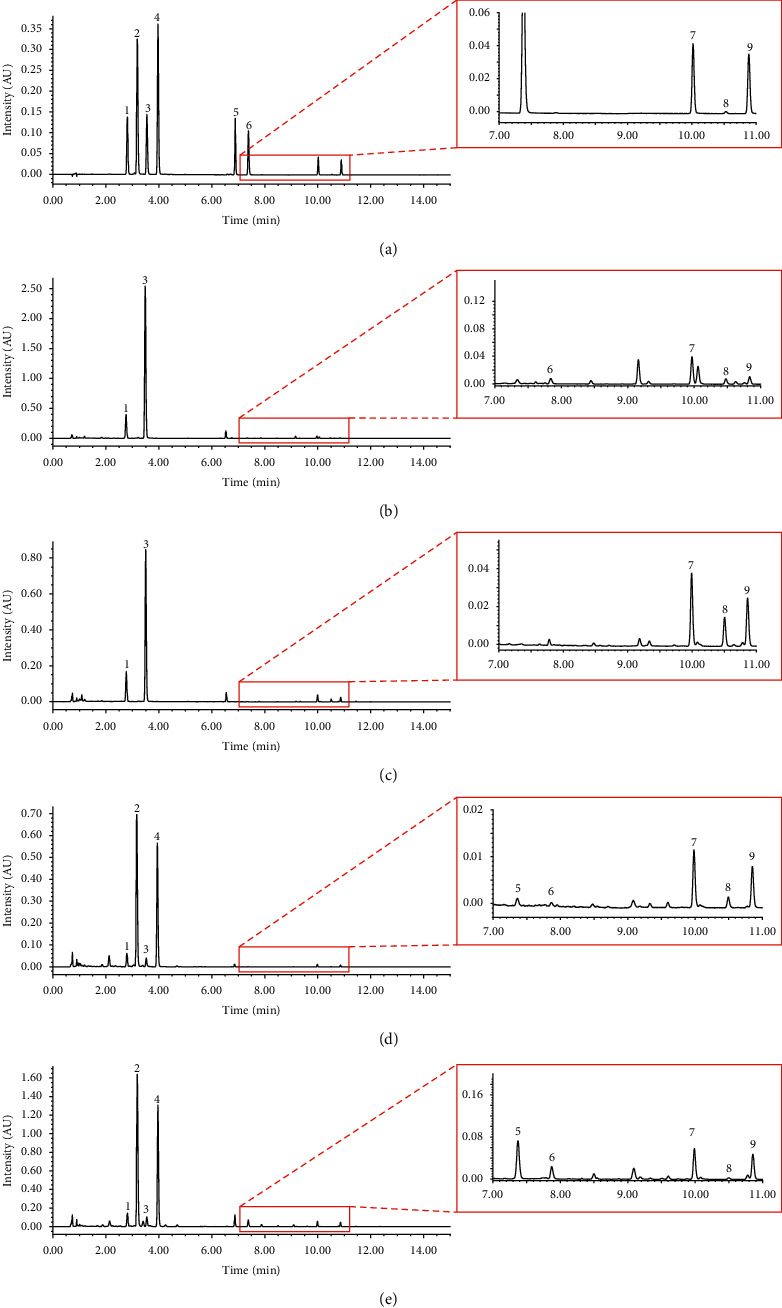
The representative UPLC chromatograms of mixed reference solution (a) and sample solutions of CRPV (b), CRP (c), AF (d), and AFI (e). (1. Nar, 2. Nan, 3. Hed, 4. Ned, 5. Por, 6. Nag, 7. Not, 8. Hep, and 9. Tar).

Flavonoids in *Citrus* CMM are shown to be associated with the treatment of liver damage, regulation of gastrointestinal motility, and other biological activities [25]. With UPLC, nine flavonoids including Nar, Nan, Hed, Ned, Por, Nag, Not, Hep, and Tar were ideally separated within 15 min (Figure 1).

In order to address the key challenge of maximizing the extraction efficiency of the tested compounds with different diverged polarities under the same extraction condition, a “spider-web” mode was preferably employed for optimizing the extraction conditions according to the multivariable valuation method presented by our group [26–30]. To express this concisely, the peak area of the tested compounds per gram was assigned as *A*_*m*-*k*_, which was correspondingly divided by their biggest peak area (*A*_*k* (max)_) to give *E*_*m*-*k*_. As shown in formula (1), *k* is denoted as different compounds, and *m* represents different extraction conditions (different solvents, different solid-liquid ratios, and different extraction times). *E*_*m-k*_ was used to construct different dimensions of the “spider-web” mode (*p*_*i*_) in formula (2). The bigger the shaded area was, the closer it was to the most optimal extraction condition. The angle between the two dimensions was tagged as *α* (*α* = 360°/*n*, *n* = 9).

(1) $$\begin{array}{c} E_{m-k}=A_{m-k}/A_{k\left(\max\right)}, \end{array}$$

(2) $$\begin{array}{c} S=\frac{1}{2}\sin\;\alpha \times \left(\sum_{i=1}^{n-1}p_{i}\cdot p_{i+1}+p_{n}\cdot p_{1}\right). \end{array}$$

**Figure 2:**
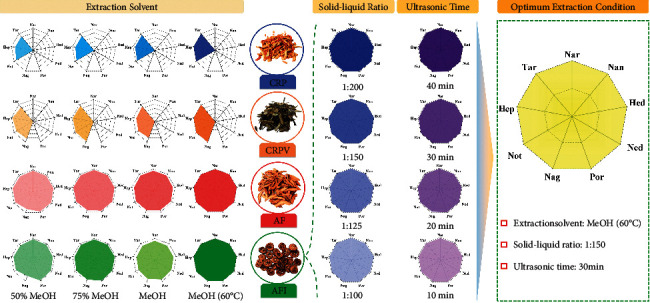
Optimization of extraction conditions by “spider-web” mode, including extraction solvent, solid-liquid ratio, and ultrasonic time.

The extraction solvents for four decoction pieces were investigated, including 50% and 75% methanol aqueous solution and methanol (60°C) (Figure 2). Under ultrasonication with methanol at 60°C, the shaded area of “spider-web” of four decoction pieces exhibited the greatest value. AFI, as a typical sample, was subjected to optimizing the solid-liquid ratio (1 : 100, 1 : 125, 1 : 150, and 1 : 200) and extraction time (10 min, 20 min, 30 min, and 40 min), to obtain optimum extraction condition. In conclusion, the best extraction was achieved by ultrasonic extraction of sample powder with methanol (solvent/material at 150 : 1) at 60°C for 30 min.

The optimized extracting method paved the way for methodological validation, whose detailed results are presented in Table 1. Intraday and interday precisions were good with relative standard deviations (RSD) less than 3.0%. The RSDs of nine tested compounds were lower than 2.8% over 12 h in the stability study at room temperature. The calibration curves of nine tested components were constructed with the determination coefficient (*r*^2^) exceeding 0.999, showing good linearity over the tested range. The LOD and LOQ values were below 0.1120 *μ*g/mL and 0.5872 *μ*g/mL, respectively. The result of the reproducibility study was satisfactory, with RSDs less than 3.0% for the tested compounds. Furthermore, the average recoveries ranged from 92.65% to 106.98% with RSD less than 2.8%. Consequently, the established method can be successfully employed in subsequent studies.

### 3.2. Quantitative Analysis of Nine Tested Compounds in Four Closely Related *Citrus* CMMs

**Figure 3:**
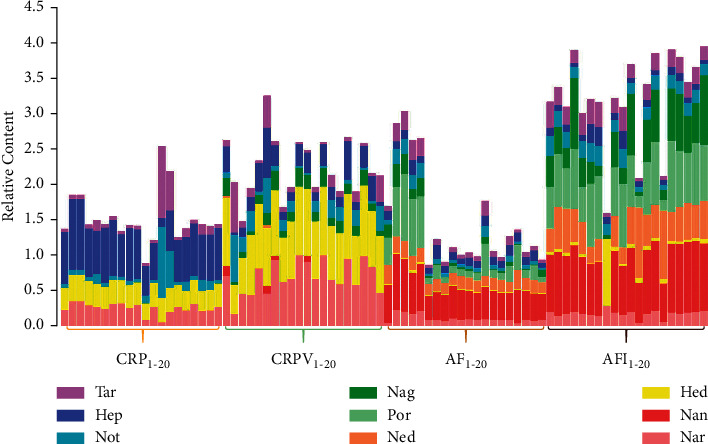
The relative content of nine flavonoids in 80 batches samples of four closely related *Citrus* CMMs.

Determined by the validated UPLC-variable wavelength method, the content of nine flavonoids in 80 batches of samples was exhibited in Table S2. In order to intuitively and clearly display the distribution of the content of interesting flavonoids in the decoction pieces, we normalized the data and expressed them by relative content, which was calculated by C_*k*_/C_*k*(max)_ (the content of the compound is divided into the maximum content of the corresponding compound, with *k* denoted as different compounds). As shown in Figure 3, the relative contents of nine flavonoids fluctuated greatly in samples from two species (*C. aurantium* L. and *C. reticulate Blanco*). The range of compounds' content can reach 2.02–46.96 mg/g for Nar (RSD, 83.99%), 1.53–186.51 mg/g for *Hed* (RSD, 109.68%), 0.22–6.91 mg/g for Not (RSD, 100.46%), 0.03–0.91 mg/g for Hep (RSD, 89.94%), and 0.10–4.21 mg/g for Tar (RSD, 110.81%), respectively. Nan, Ned, and Por were not detected in the pericarp of C. *reticulate Blanco*, whose contents in the fruit of C. *aurantium* L. were in the range of 0.52–117.24 mg/g (RSD, 40.16%), 0.57–195.73 mg/g (RSD, 65.07%), and 0.00–5.16 mg/g (RSD, 68.45%), respectively. The content of Nag in samples was between 0.02 and 3.17 mg/g (RSD, 106.86%) except for CRP.

The characteristics of components' accumulation in the two species at different development stages were studied. In the fruit of C. *aurantium* L., the contents of the tested components were higher in AFI than in AF except for Hep, and the total content in AFI was significantly higher (the average value was 219.36 mg/g) than that in AF (the average value was 101.87 mg/g). The result suggests that as fruits become ripe, the total content of flavonoids decreases correspondingly. Similarly, in the pericarp of C. *reticulate Blanco*, the total content of focused analytes can be up to 189.39 mg/g in CRPV, while 73.76 mg/g was measured in CRP. This may be due to the different phenotypes of secondary metabolites in samples at different maturation stages [21, 22]. Generally, the content of flavonoids in unripe samples (AFI or CRPV) was more abundant than that in ripe samples (AF or CRP).

### 3.3. Discrimination of Closely Related *Citrus* CMMs by Chemometrics

As one of the main tools of multivariate statistical methods, principal component analysis (PCA) is usually used to set up a low-dimensional plane or space that can visualize the classification trends among samples [31, 32]. It has also been used to remove outliers from data [33]. By introducing an orthogonal signal correction (OSC) filter, variation from *X* (descriptor variables) was removed to reduce model complexity by orthogonal projection to latent structure-discriminate analysis (OPLS-DA), which is uncorrelated with *Y* (property variables) to achieve good discrimination for samples [34, 35]. In addition, it has always been successfully applied to study metabolomics and CMMs [36–39].

**Figure 4:**
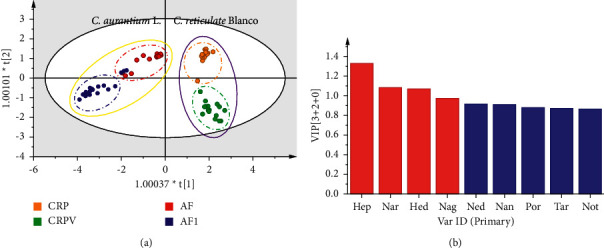
Discrimination of four closely related *Citrus* CMMs by OPLS-DA (a), and VIP values of the tested compounds (b). The compounds were marked as red, which had important effects on the differentiation of samples.

For ensuring the accuracy of the model, outliers (AFI8, AF4, CRPV2, CRPV6, CRP11, and CRP13) were removed before the construction of the distinguishing model by PCA (Figure S1). As shown in Figure 4(a), AFI, AF, CRPV, and CRP were successfully discriminated by the first principal component (PC1) and second principal component (PC2), which accounted for 52.4% and 16.2%, respectively. The good fit of the OPLS-DA model was displayed by *R*^2^*X* (0.976) and *R*^2^*Y* (0.886). The OPLS-DA model had a goodness-of-prediction (*Q*^2^ = 0.864). For assessing the prediction capability, cross-validation of the OPLS-DA models was performed (Figure S2). The obtained results proved that the model was established validly. As shown in Figure 4(b), Hep, Nar, Hed, and Nag were filtered out by variable importance for the projection (VIP) value (VIP > 1.0), which were regarded as chemotaxonomic markers for discriminating four closely related *Citrus* CMMs.

**Figure 5:**
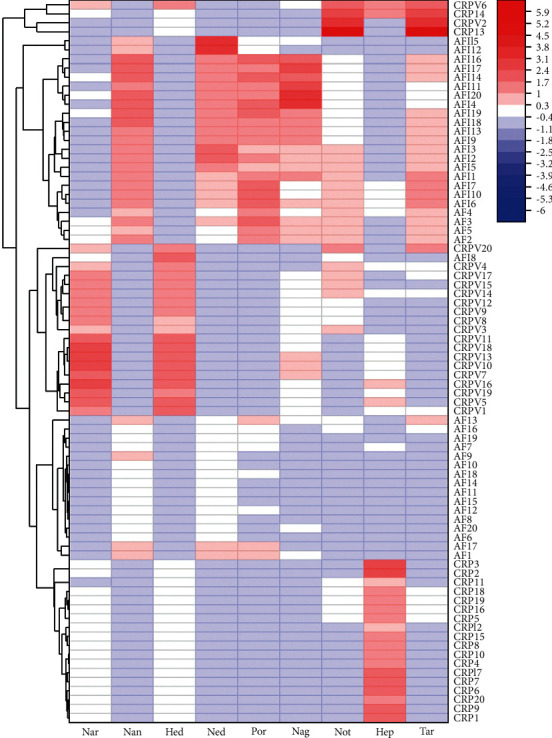
Clustering heatmap analysis based on flavonoids' content in four decoction pieces.

Furthermore, a clustering heatmap was applied to show the trend in the relative content of flavonoids and the relationship of the classified samples (Figure 5). A red box indicates that the content occurs at higher levels compared to the mean level in a sample, while a blue box means that the content is lower. Meanwhile, the classified clusters were consistent with the results of OPLS-DA. Four closely related *Citrus* CMMs were well distinguished. Collectively, chemometrics was proved as a feasible strategy to discriminate closely related genus CMMs.

### 3.4. Analysis of Chemotaxonomic Markers by Network Pharmacological

As one popular strategy, ingenuity pathway analysis (IPA) can be used to predict correlations among components, targets, and diseases through unique algorithms and reported literature [40]. Focusing on screened chemotaxonomic markers including Hep, Nar, Hed, and Nag, a network of components-targets-diseases was constructed to understand the quality differences of closely related *Citrus* CMMs.

**Figure 6:**
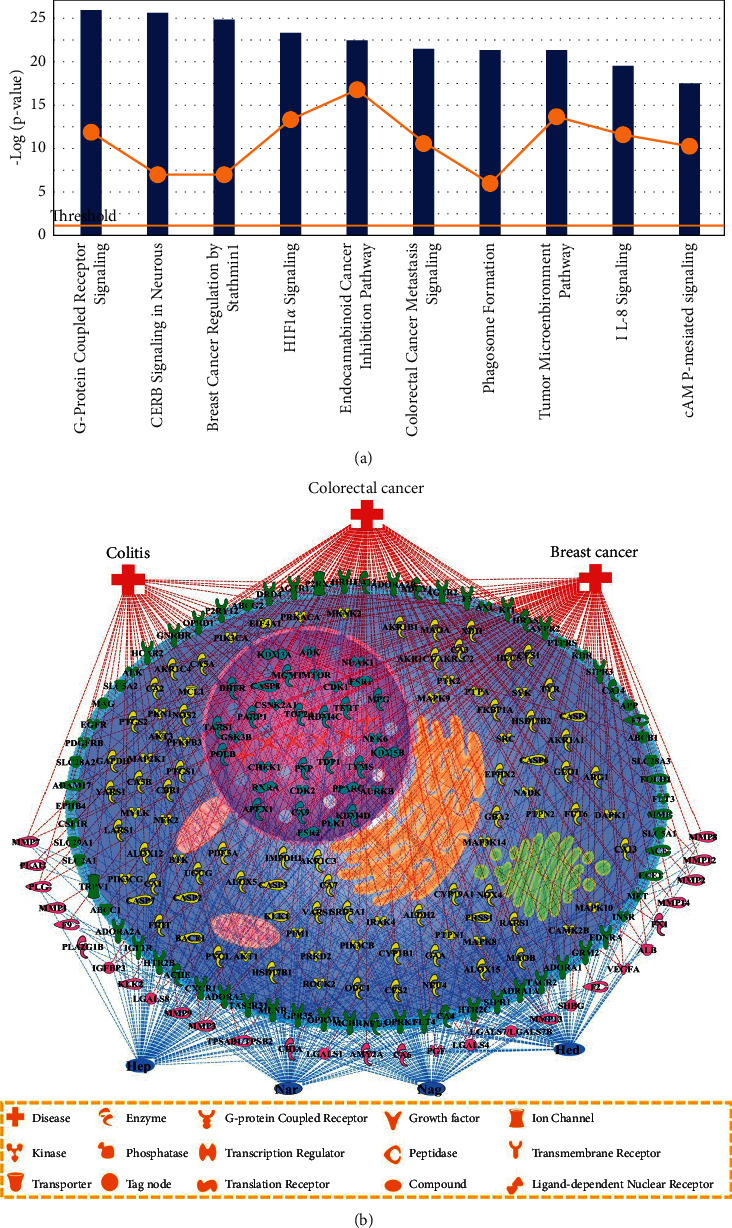
Top 10 pathways in targets associated chemotaxonomic markers (a). Network diagram of components-targets-diseases (b). (Blue represents the chemotaxonomic markers, red represents the disease, and other colors represent the targets in different locations).

Using the PubChem database, we obtained the structures of Hep, Nar, Hed, and Nag. In the Swiss Target Prediction database, 232 targets (Table S4) were identified by the correspondence with Hep, Nar, Hed, and Nag, after removing the redundant targets with *Homo* as a limited species. Then, 223 targets were focused on by IPA for core analysis. Furthermore, 465 canonical pathways and 21 networks were illustrated, revealing the close associations between targets and inflammation, cancer, and neurological disorders. As shown in Figure 6(a), the yellow threshold line indicates *p*=0.05. The ranking of the relationship between targets and pathways revealed that the regulation of these targets by the tested compounds may respectively exhibit anti-inflammatory, anticancer, and neuroprotective activities through IL-8 signaling pathway, colorectal cancer metastasis signaling pathway, and CERB signaling in neural. The related diseases, including breast cancer, colorectal cancer, and colitis, were selected for the construction of components-targets-diseases network. Four compounds, 223 targets, and three diseases were combined by using the function of “pathway designer”. As shown in Figure 6(b), 126 targets were related to breast cancer, 148 targets were associated with colorectal cancer, and 54 targets were involved in colitis, respectively. It is speculated that Hep, Nar, Hed, and Nag exert induction effects on the treatment of colitis, breast cancer, and colorectal cancer, which is consistent with some reported literature [41–43].

## 4. Conclusions

In the presented work, the established UPLC-variable wavelength detection method showed acceptable linearity, precision, repeatability, and accuracy, which was successfully used for quantitative analysis of nine major flavonoids in four closely related *Citrus* CMMs. The total content of flavonoids fluctuated obviously in different species (C. *aurantium* L. and C. *reticulate Blanco*) with a decrease in both species at maturity. Meanwhile, OPLS-DA and cluster analysis were employed to perform excellent discrimination of AFI, AF, CRPV, and CRP. By OPLS-DA, the screened compounds with VIP values (VIP > 1.0) were suggested as four chemotaxonomic markers (Hep, Nar, Hed, and Nag). Furthermore, a components-targets-diseases network was developed to display the correlation between chemotaxonomic markers and diseases of colitis, breast cancer, and colon cancer via the focused targets. This study firstly presents a comprehensive strategy for simultaneous comparison of four closely related *Citrus* CMMs based on the flavonoids' content, which may offer a simple and convenient method for studying closely related genus CMMs.

## Figures and Tables

**Table 1:** Summary result of linear regression, LODs, LOQs, precision, repeatability, stability, and recovery for the tested compounds in AFI.

Compounds	Linear regression			LODs (*μ*g/mL)	LOQs (*μ*g/mL)	Precision		Repeatability (*n* = 6, RSD (%))	Stability (*n* = 7, RSD (%))	Recovery (*n* = 6, mean ± SD (%))
	Regression equation	*r* ^2^	Linear range (*μ*g/mL)			Intraday (*n* = 6, RSD (%))	Inter-day (*n* = 3, RSD (%))			
Nar	*y* = 6,948.0 *x* + 5,600.7	0.999	9.074–580.7	0.1120	0.3360	1.6	1.7	0.5	2.0	94.96 ± 1.23
Nan	*y* = 6,772,8 *x* + 75,181.5	0.999	20.62–1320	0.0849	0.2546	2.4	2.3	1.9	1.6	98.58 ± 2.74
Hed	*y* = 7604.3 *x* + 7644.4	0.999	8.935–571.8	0.1103	0.3309	1.2	1.1	0.8	1.7	92.65 ± 2.11
Ned	*y* = 7,311.8 *x* + 109,568.9	0.999	20.32–1301	0.0836	0.2509	2.4	2.4	2.3	1.7	97.07 ± 2.75
Por	*y* = 6575.2 *x* + 2079.1	0.999	5.285–338.2	0.0652	0.5872	1.9	2.1	1.3	2.8	106.98 ± 1.16
Nag	*y* = 13,451.8 *x* + 3,874.2	0.999	3.292–210.7	0.0406	0.1219	2.8	2.4	0.9	2.8	97.00 ± 1.23
Not	*y* = 15,512.3 *x* + 519.9	0.999	0.944–60.44	0.0350	0.1049	2.4	2.6	1.2	2.1	101.25 ± 1.50
Hep	*y* = 11,381.8 *x* + 314.0	0.999	0.040–2.545	0.0398	0.1591	2.8	2.6	1.6	2.7	93.50 ± 2.09
Tar	*y* = 16,347.1 *x* + 738.7	0.999	0.786–50.29	0.0291	0.0873	2.2	1.9	0.6	1.9	94.76 ± 1.51

## Data Availability

The data used to support the findings of this study are available from the corresponding author upon request.

## Supplementary Materials

Supplementary Materials Table S1: sample information of four closely related *Citrus* CMMs. Table S2: the content of nine flavonoids in the four closely related *Citrus* CMMs samples (mg/g, *n* = 3), and “–”, signal below LOD. Table S3: all the targets corresponding to four chemotaxonomic markers. Figure S1: outlier identified by PCA score from AFI (a), AF (b), CRPV (c), and CRP (d), respectively. Red circles indicate abnormal samples. Figure S2: a permutations test (*n* = 200) performed to give [*R*^2^  = (0.0, 0.0107), *Q*^2^ = (0.0, −0.202)] for AFI (a), [*R*^2^ = (0.0, 0.0153), *Q*^2^ = (0.0, −0.196)] for AF (b), [*R*^2^ = (0.0, 0.000873), *Q*^2^ = (0.0, −0.218)] for CRPV (c), and [*R*^2^ = (0.0, 0.0089), *Q*^2^ = (0.0, −0.21)] for CRP (d) in the OPLS-DA.
