# Supplementary material for: Study on Closely Related Citrus CMMs based on Chemometrics and Prediction of Components-Targets-Diseases Network by Ingenuity Pathway Analysis
Source: Evid Based Complement Alternat Med. 2022 Mar 31;2022:1106353. doi: 10.1155/2022/1106353 (PMC9068285; doi:10.1155/2022/1106353)

**Supplementary Materials to:**

**Study on** **closely**-**related *Citrus* CMMs based on chemometrics and prediction of components**-**targets**-**diseases network by Ingenuity Pathway Analysis**

Qixuan Mu^1^, Yaping Zhang^1^, Ying Cui^1^, Xin Chai^1^, Junlian Liu^2^, Yongzhi Li^2^*, Huijuan Yu^1^*, Yuefei Wang^1^

^1^ State Key Laboratory of Component-based Chinese Medicine, Tianjin Key Laboratory of TCM Chemistry and Analysis, Tianjin University of Traditional Chinese Medicine, Tianjin 301617, China.

^2^ China Astronaut Research and training center, Beijing 100094, China.

**^*^**Correspondence should be addressed to Yongzhi Li; liyongzhi666@sina.com; Huijuan Yu; huijuanyu@tjutcm.edu.cn

Table S1: Sample information of four closely-related *Citrus* CMMs.

| Batch No. | Habitat | Batch  No. | Habitat | Batch  No | Habitat | Batch  No | Habitat |
| --- | --- | --- | --- | --- | --- | --- | --- |
| AFI1 | Hunan | AF1 | Jiangxi | CRP1 | Hunan | CRPV1 | Zhejiang |
| AFI2 | Hunan | AF2 | Jiangxi | CRP2 | Hunan | CRPV2 | Zhejiang |
| AFI3 | Hunan | AF3 | Hunan | CRP3 | Hunan | CRPV3 | Hunan |
| AFI4 | Hunan | AF4 | Hunan | CRP4 | Hunan | CRPV4 | Hunan |
| AFI5 | Hunan | AF5 | Hunan | CRP5 | Hunan | CRPV5 | Hunan |
| AFI6 | Hunan | AF6 | Hunan | CRP6 | Zhejiang | CRPV6 | Hunan |
| AFI7 | Hunan | AF7 | Jiangxi | CRP7 | Zhejiang | CRPV7 | Sichuan |
| AFI8 | Jiangxi | AF8 | Jiangxi | CRP8 | Zhejiang | CRPV8 | Sichuan |
| AFI9 | Jiangxi | AF9 | Jiangxi | CRP9 | Zhejiang | CRPV9 | Jiangxi |
| AFI10 | Jiangxi | AF10 | Jiangxi | CRP10 | Zhejiang | CRPV10 | Sichuan |
| AFI11 | Hunan | AF11 | Jiangxi | CRP11 | Sichuan | CRPV11 | Hunan |
| AFI12 | Sichuan | AF12 | Jiangxi | CRP12 | Zhejiang | CRPV12 | Zhejiang |
| AFI13 | Jiangxi | AF13 | Jiangxi | CRP13 | Zhejiang | CRPV13 | Hunan |
| AFI14 | Hunan | AF14 | Jiangxi | CRP14 | Hubei | CRPV14 | Hunan |
| AFI15 | Jiangxi | AF15 | Jiangxi | CRP15 | Guangdong | CRPV15 | Jiangxi |
| AFI16 | Jiangxi | AF16 | Hunan | CRP16 | Sichuan | CRPV16 | Sichuan |
| AFI11 | Jiangxi | AF17 | Jiangxi | CRP17 | Sichuan | CRPV17 | Hunan |
| AFI18 | Jiangxi | AF18 | Jiangxi | CRP18 | Sichuan | CRPV18 | Jiangxi |
| AFI19 | Jiangxi | AF19 | Jiangxi | CRP19 | Sichuan | CRPV19 | Sichuan |
| AFI20 | Jiangxi | AF20 | Hunan | CRP20 | Zhejiang | CRPV20 | Sichuan |

Table S2: The content of nine flavonoids in four closely-related *Citrus* CMMs (mg/g, *n* = 3)

| Batch | Nar | Nan | Hed | Ned | Por | Nag | Not | Hep | Tar |
| --- | --- | --- | --- | --- | --- | --- | --- | --- | --- |
| CRP1 | 11.11 | – | 57.63 | – | – | – | 0.35 | 0.67 | 0.15 |
| CRP2 | 16.67 | – | 68.90 | – | – | – | 0.52 | 0.91 | 0.21 |
| CRP3 | 16.67 | – | 68.90 | – | – | – | 0.52 | 0.91 | 0.21 |
| CRP4 | 14.12 | – | 63.76 | – | – | – | 0.39 | 0.62 | 0.18 |
| CRP5 | 13.12 | – | 59.44 | – | – | – | 0.99 | 0.55 | 0.63 |
| CRP6 | 11.62 | – | 57.84 | – | – | – | 0.36 | 0.72 | 0.16 |
| CRP7 | 14.91 | – | 62.37 | – | – | – | 0.38 | 0.72 | 0.18 |
| CRP8 | 15.26 | – | 61.87 | – | – | – | 0.31 | 0.54 | 0.15 |
| CRP9 | 12.34 | – | 60.81 | – | – | – | 0.34 | 0.68 | 0.15 |
| CRP10 | 14.16 | – | 59.02 | – | – | – | 0.37 | 0.63 | 0.19 |
| CRP11 | 4.19 | – | 43.84 | – | – | – | 0.75 | 0.38 | 0.13 |
| CRP12 | 12.91 | – | 62.75 | – | – | – | 0.31 | 0.47 | 0.14 |
| CRP13 | 2.72 | – | 64.49 | – | – | – | 6.91 | 0.11 | 4.21 |
| CRP14 | 9.89 | – | 61.41 | – | – | – | 3.62 | 0.51 | 2.34 |
| CRP15 | 13.05 | – | 56.83 | – | – | – | 0.32 | 0.54 | 0.14 |
| CRP16 | 10.62 | – | 51.10 | – | – | – | 0.90 | 0.58 | 0.45 |
| CRP17 | 14.89 | – | 62.75 | – | – | – | 0.38 | 0.67 | 0.19 |
| CRP18 | 10.56 | – | 52.13 | – | – | – | 1.04 | 0.58 | 0.56 |
| CRP19 | 10.68 | – | 53.13 | – | – | – | 0.87 | 0.60 | 0.46 |
| CRP20 | 13.03 | – | 60.70 | – | – | – | 0.38 | 0.66 | 0.19 |
| CRPV1 | 33.53 | 16.46 | 179.12 | 4.43 | – | 0.80 | 0.56 | 0.33 | 0.35 |
| CRPV2 | 8.37 | 0.23 | 75.79 | 0.34 | – | 0.12 | 4.57 | 0.03 | 2.95 |
| CRPV3 | 21.24 | – | 95.57 | – | – | 0.26 | 1.44 | 0.13 | 0.31 |
| CRPV4 | 20.62 | – | 158.48 | – | – | 0.16 | 1.99 | 0.18 | 0.47 |
| CRPV5 | 38.40 | – | 168.61 | – | – | 0.43 | 0.30 | 0.37 | 0.11 |
| CRPV6 | 21.69 | 12.35 | 152.12 | 8.37 | – | 0.56 | 3.40 | 0.64 | 1.87 |
| CRPV7 | 44.14 | 6.24 | 172.92 | – | – | 0.88 | 0.44 | 0.27 | 0.22 |
| CRPV8 | 29.47 | – | 117.08 | – | – | 0.30 | 1.02 | 0.10 | 0.28 |
| CRPV9 | 31.48 | – | 150.70 | – | – | 0.42 | 1.06 | 0.11 | 0.28 |
| CRPV10 | 46.96 | – | 181.17 | – | – | 0.82 | 0.22 | 0.27 | 0.10 |
| CRPV11 | 42.87 | 9.81 | 176.50 | – | – | 0.60 | 0.22 | 0.26 | 0.10 |
| CRPV12 | 31.48 | – | 150.70 | – | – | 0.42 | 1.06 | 0.11 | 0.28 |
| CRPV13 | 46.96 | – | 181.17 | – | – | 0.82 | 0.22 | 0.27 | 0.10 |
| CRPV14 | 30.57 | – | 143.81 | – | – | 0.35 | 1.72 | 0.17 | 0.70 |
| CRPV15 | 27.83 | – | 136.36 | – | – | 0.50 | 1.38 | 0.13 | 0.33 |
| CRPV16 | 44.89 | – | 170.71 | – | – | 0.45 | 0.39 | 0.50 | 0.19 |
| CRPV17 | 27.63 | – | 129.43 | – | – | 0.43 | 1.55 | 0.10 | 0.59 |

Table S2 continued

| CRPV18 | 46.46 | – | 186.51 | – | – | 0.69 | 0.23 | 0.28 | 0.11 |
| --- | --- | --- | --- | --- | --- | --- | --- | --- | --- |
| CRPV19 | 39.33 | – | 146.65 | – | – | 0.34 | 0.28 | 0.32 | 0.12 |
| CRPV20 | 22.06 | – | 150.58 | – | – | 0.21 | 2.47 | 0.05 | 1.56 |
| AF1 | 2.41 | 62.19 | 2.19 | 53.52 | 2.00 | 0.57 | 0.42 | 0.12 | 0.26 |
| AF2 | 10.93 | 92.90 | 2.91 | 45.06 | 3.57 | 1.03 | 1.44 | 0.10 | 1.07 |
| AF3 | 9.90 | 86.78 | 3.89 | 45.93 | 4.85 | 0.92 | 1.39 | 0.12 | 1.10 |
| AF4 | 8.31 | 67.29 | 7.16 | 39.43 | 4.16 | 0.42 | 1.40 | 0.19 | 1.13 |
| AF5 | 10.31 | 77.90 | 3.33 | 40.29 | 3.75 | 0.83 | 1.44 | 0.09 | 0.99 |
| AF6 | 4.44 | 39.28 | 2.49 | 29.86 | 0.43 | 0.09 | 0.26 | 0.07 | 0.18 |
| AF7 | 4.44 | 44.88 | 2.62 | 33.81 | 1.03 | 0.04 | 0.52 | 0.17 | 0.34 |
| AF8 | 3.48 | 43.23 | 2.23 | 30.81 | 0.40 | 0.03 | 0.33 | 0.09 | 0.22 |
| AF9 | 5.41 | 52.82 | 2.52 | 33.79 | 0.52 | 0.12 | 0.36 | 0.09 | 0.26 |
| AF10 | 4.21 | 49.60 | 2.35 | 35.99 | 0.53 | 0.05 | 0.29 | 0.08 | 0.20 |
| AF11 | 4.73 | 47.20 | 2.66 | 33.65 | 0.48 | 0.06 | 0.38 | 0.10 | 0.26 |
| AF12 | 4.31 | 43.82 | 2.33 | 33.19 | 0.58 | 0.02 | 0.35 | 0.10 | 0.21 |
| AF13 | 4.70 | 53.59 | 1.53 | 27.70 | 2.39 | 0.30 | 1.03 | 0.13 | 0.85 |
| AF14 | 4.26 | 47.40 | 2.49 | 35.10 | 0.54 | 0.03 | 0.41 | 0.11 | 0.29 |
| AF15 | 4.45 | 43.88 | 2.60 | 33.59 | 0.52 | 0.07 | 0.31 | 0.09 | 0.20 |
| AF16 | 4.41 | 44.18 | 2.12 | 29.02 | 1.16 | 0.16 | 0.64 | 0.14 | 0.48 |
| AF17 | 2.02 | 55.35 | 1.98 | 52.65 | 1.82 | 0.04 | 0.26 | 0.12 | 0.14 |
| AF18 | 4.24 | 47.83 | 2.29 | 33.98 | 0.59 | 0.03 | 0.36 | 0.11 | 0.26 |
| AF19 | 3.84 | 44.71 | 2.39 | 34.10 | 1.09 | 0.03 | 0.36 | 0.14 | 0.22 |
| AF20 | 4.46 | 42.07 | 2.50 | 29.94 | 0.41 | 0.30 | 0.25 | 0.06 | 0.17 |
| AFI1 | 9.58 | 95.05 | 5.98 | 65.38 | 3.04 | 1.36 | 1.98 | 0.11 | 1.52 |
| AFI2 | 6.80 | 105.81 | 7.69 | 117.08 | 3.84 | 1.21 | 1.54 | 0.08 | 1.06 |
| AFI3 | 8.23 | 95.65 | 9.21 | 121.17 | 2.95 | 0.91 | 1.57 | 0.09 | 1.04 |
| AFI4 | 9.51 | 110.22 | 8.94 | 90.82 | 4.39 | 3.17 | 1.05 | 0.05 | 0.74 |
| AFI5 | 8.55 | 93.96 | 7.36 | 88.94 | 2.54 | 1.28 | 1.68 | 0.07 | 1.27 |
| AFI6 | 7.54 | 83.73 | 2.37 | 46.86 | 4.59 | 0.96 | 1.82 | 0.25 | 1.45 |
| AFI7 | 7.11 | 89.23 | 3.15 | 60.49 | 4.57 | 0.43 | 1.96 | 0.24 | 1.47 |
| AFI8 | 13.59 | 0.52 | 174.98 | 0.57 | – | 0.18 | 1.06 | 0.09 | 0.22 |
| AFI9 | 9.24 | 102.44 | 10.56 | 83.76 | 3.56 | 1.57 | 1.19 | 0.09 | 0.88 |
| AFI10 | 7.57 | 80.51 | 5.04 | 47.03 | 4.61 | 0.73 | 1.78 | 0.24 | 1.41 |
| AFI11 | 9.54 | 106.11 | 10.87 | 101.89 | 3.76 | 2.75 | 1.07 | 0.06 | 0.77 |
| AFI12 | 2.37 | 65.58 | 12.22 | 195.73 | 0.86 | 0.17 | 0.45 | 0.08 | 0.17 |
| AFI13 | 8.16 | 106.48 | 10.06 | 86.20 | 3.84 | 1.89 | 1.21 | 0.10 | 0.90 |
| AFI14 | 10.43 | 115.33 | 8.42 | 90.92 | 4.54 | 2.36 | 1.35 | 0.07 | 0.99 |
| AFI15 | 3.02 | 63.15 | 11.54 | 189.22 | 0.77 | 0.46 | 0.46 | 0.07 | 0.19 |
| AFI16 | 9.28 | 113.70 | 6.18 | 80.24 | 5.16 | 2.36 | 1.33 | 0.10 | 1.01 |

Table S2 continued

| AFI17 | 8.24 | 115.89 | 6.15 | 95.04 | 4.10 | 2.54 | 1.30 | 0.09 | 0.95 |
| --- | --- | --- | --- | --- | --- | --- | --- | --- | --- |
| AFI18 | 9.23 | 116.27 | 6.98 | 100.19 | 3.69 | 1.62 | 1.24 | 0.08 | 0.90 |
| AFI19 | 9.85 | 117.24 | 7.01 | 90.28 | 4.50 | 1.81 | 1.30 | 0.07 | 0.96 |
| AFI20 | 10.41 | 111.33 | 11.11 | 105.85 | 4.06 | 3.11 | 1.10 | 0.06 | 0.76 |

“–”, Signal below LOD.

Table S3: All the targets corresponding to four chemotaxonomic markers.

| Targets | | | |
| --- | --- | --- | --- |
| ABCB1 | CDK2 | KIT | PKN1 |
| ABCC1 | CES1 | KLK1 | PLA2G1B |
| ABCG2 | CES2 | KLK2 | PLAU |
| ACE | CHEK1 | LARS1 | PLG |
| ACHE | CHIA | LGALS1 | PLK1 |
| ADAM17 | CSF1R | LGALS4 | PNP |
| ADCY1 | CSNK2A1 | LGALS7/LGALS7B | POLB |
| ADK | CXCR1 | LGALS8 | PPARG |
| ADORA1 | CYP19A1 | MAG | PRKACA |
| ADORA2A | CYP1B1 | MAOA | PRKD2 |
| ADORA2B | DAPK1 | MAOB | PRSS1 |
| ADORA3 | DHFR | MAP2K1 | PTGS1 |
| ADRA1A | DRD4 | MAP3K14 | PTGS2 |
| AGTR1 | ECE1 | MAPK10 | PTK2 |
| AGTR2 | EDNRA | MAPK8 | PTPA |
| AKR1A1 | EGFR | MAPK9 | PTPN1 |
| AKR1B1 | EIF4A1 | MCHR1 | PTPN2 |
| AKR1C1/AKR1C2 | EPHB4 | MCL1 | PTPRS |
| AKR1C3 | EPHX2 | MET | PYGL |
| AKR1C4 | ESR1 | MGMT | RARS1 |
| AKT1 | ESR2 | MKNK2 | ROCK2 |
| AKT3 | F2 | MLNR | RXRA |
| ALB | F3 | MME | S1PR1 |
| ALDH2 | F7 | MMP1 | S1PR3 |
| ALK | F9 | MMP12 | SHBG |
| ALOX12 | FHIT | MMP13 | SLC28A2 |
| ALOX15 | FKBP1A | MMP14 | SLC28A3 |
| ALOX5 | FLT3 | MMP2 | SLC29A1 |
| AMY2A | FLT4 | MMP3 | SLC2A1 |
| APEX1 | FN1 | MMP7 | SLC5A1 |
| APP | FOLH1 | MMP8 | SLC5A2 |
| ARG1 | FUT6 | MMP9 | SRC |
| AURKB | GAA | MPG | SRD5A1 |
| AVPR2 | GAPDH | MTOR | SYK |
| AXL | GBA2 | MYLK | TACR2 |
| BACE1 | GLO1 | NADK | TARS1 |
| BTK | GNRHR | NEK2 | TAS2R31 |
| CA1 | GPR35 | NEK6 | TDP1 |
| CA12 | GRM2 | NEU3 | TERT |
| CA13 | GSK3B | NEU4 | TOP2A |
| CA14 | HCAR2 | NOS2 | TPSAB1/TPSB2 |
| CA2 | HLCS | NOX4 | TRPV1 |

Table S3 continued

| Targets | | | |
| --- | --- | --- | --- |
| CA3 | HRAS | NUAK1 | TYMS |
| CA4 | HRH3 | ODC1 | TYR |
| CA5A | HSD17B1 | OPRD1 | UGCG |
| CA5B | HSD17B2 | OPRK1 | VARS1 |
| CA6 | HTR2B | OPRM1 | VEGFA |
| CA7 | HTR2C | P2RX3 | XDH |
| CA9 | IGF1R | P2RY12 | CDK1 |
| CAMK2B | IGFBP3 | PARP1 | KDR |
| CASP1 | IMPDH1 | PDE5A | PIM1 |
| CASP2 | INSR | PDGFRB | PIK3CG |
| CASP3 | IRAK4 | PFKFB3 | KDM5B |
| CASP6 | KDM3A | PGF | CBR1 |
| CASP7 | KDM4C | PIK3CA | PIK3CB |
| CASP8 | KDM4D |  |  |


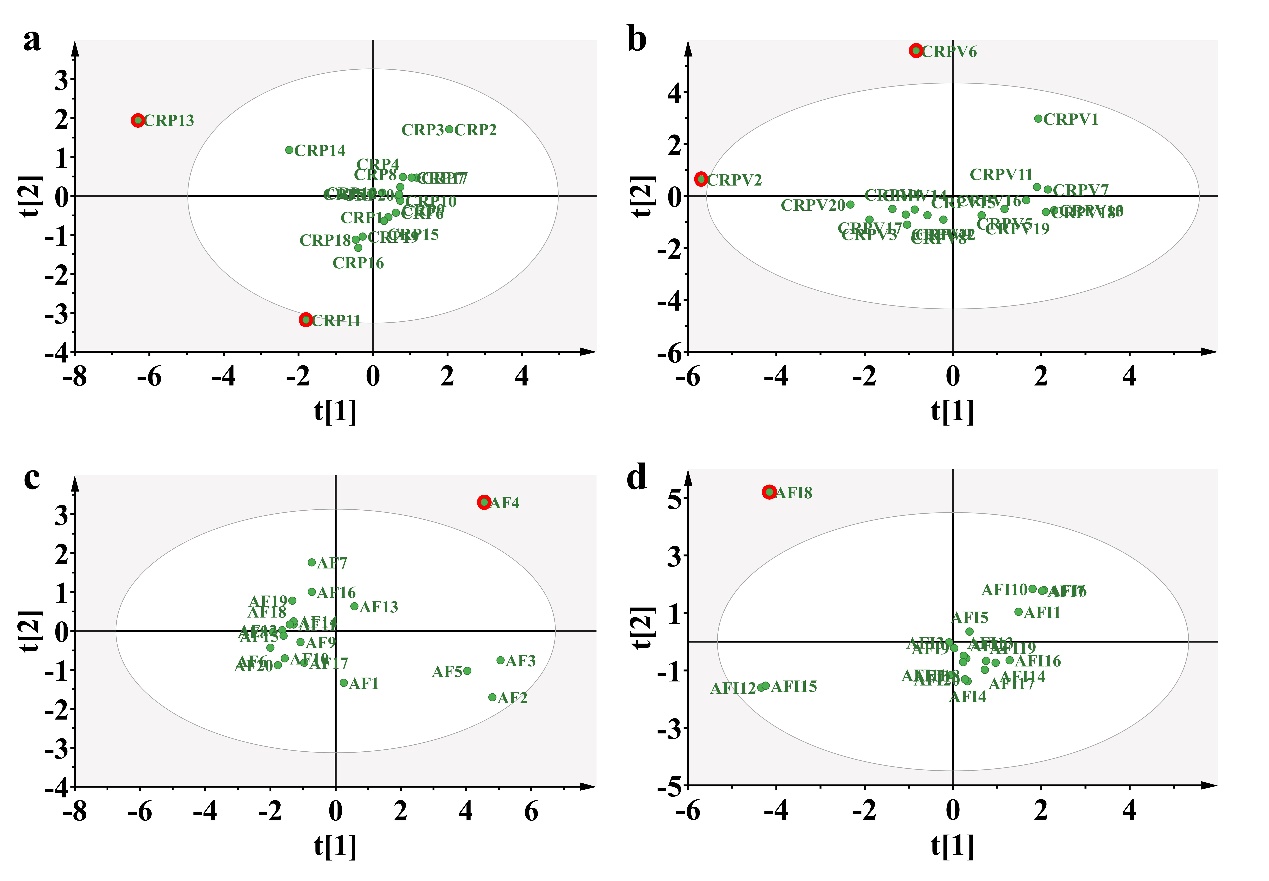


Figure S1: Outlier identified by PCA score from AFI (a), AF (b), CRPV (c), and CRP (d), respectively. Red circles indicate abnormal samples.


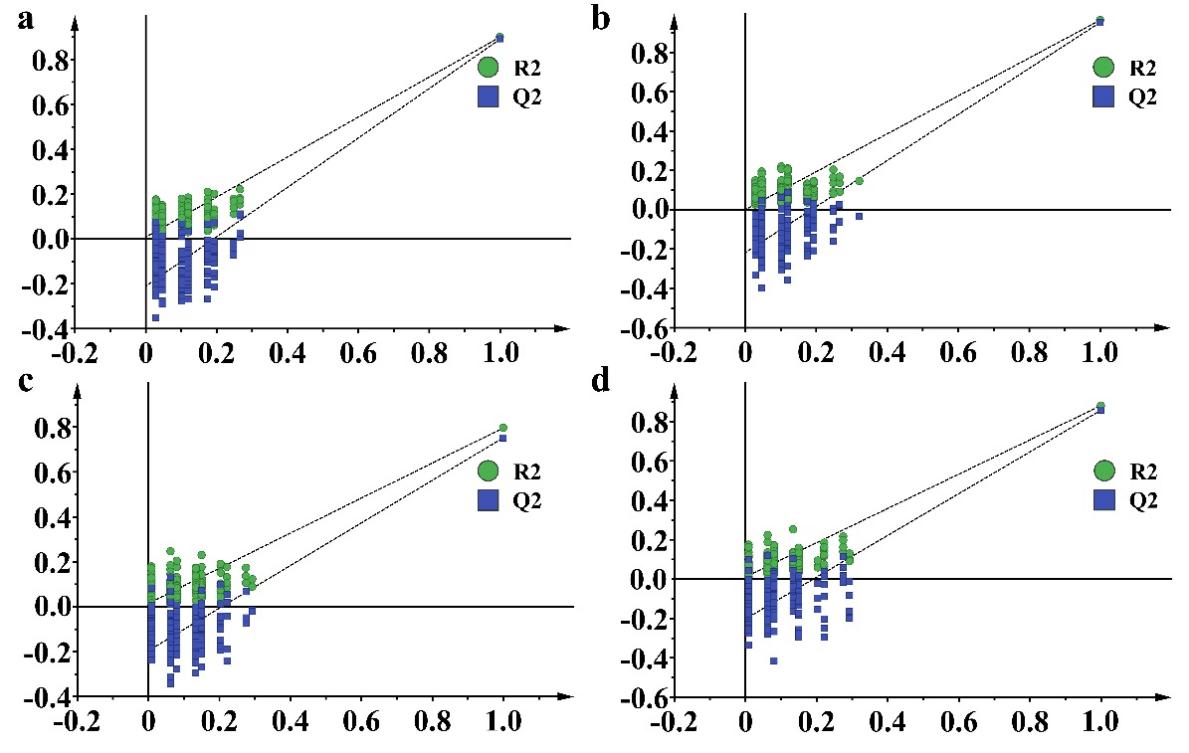


Figure S2: A permutations test (n = 200) performed to give [R^2^ = (0.0, 0.0107), Q^2^ = (0.0, −0.202)] for AFI (a), [R^2^ = (0.0, 0.0153), Q^2^ = (0.0, −0.196)] for AF (b), [R^2^= (0.0, 0.000873), Q^2^ = (0.0, −0.218)] for CRPV (c), and [R^2^ = (0.0, 0.0089), Q^2^ = (0.0, −0.21)] for CRP (d) in the OPLS-DA.

Graphical Abstract


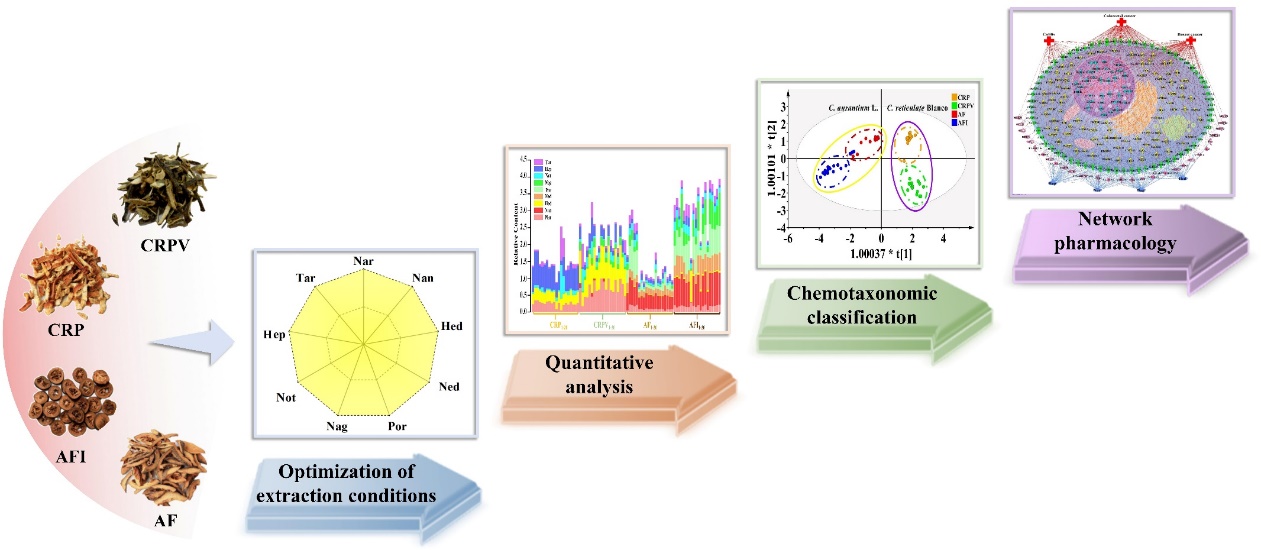

Supplement: Supplementary Materials — Table S1: sample information of four closely related Citrus CMMs. Table S2: the content of nine flavonoids in the four closely related Citrus CMMs samples (mg/g, n = 3), and “–”, signal below LOD. Table S3: all the targets corresponding to four chemotaxonomic markers. Figure S1: outlier identified by PCA score from AFI (a), AF (b), CRPV (c), and CRP (d), respectively. Red circles indicate abnormal samples. Figure S2: a permutations test (n = 200) performed to give [R2 = (0.0, 0.0107), Q2 = (0.0, −0.202)] for AFI (a), [R2 = (0.0, 0.0153), Q2 = (0.0, −0.196)] for AF (b), [R2 = (0.0, 0.000873), Q2 = (0.0, −0.218)] for CRPV (c), and [R2 = (0.0, 0.0089), Q2 = (0.0, −0.21)] for CRP (d) in the OPLS-DA. [file 1106353.f1.docx]
